# Case Report: 40Hz multi-target transcranial alternating current stimulation combined with rehabilitation for post-stroke cognitive impairment

**DOI:** 10.3389/fpsyt.2025.1682068

**Published:** 2025-10-20

**Authors:** Ming Hui Lai, Yi Fan Wang, Yan Lu, Wang Fu, En Bang Zhang, Hong Li Ma, Chun Lei Shan, Feng Wang, Shang Jun Huang, Cong Wang, Xiao Ming Yu

**Affiliations:** ^1^ Department of Rehabilitation, Seventh People’s Hospital of Shanghai University of Traditional Chinese Medicine, Shanghai, China; ^2^ Department of Neurology, Seventh People’s Hospital of Shanghai University of Traditional Chinese Medicine, Shanghai, China; ^3^ Department of Imaging, Seventh People’s Hospital of Shanghai University of Traditional Chinese Medicine, Shanghai, China; ^4^ Engineering Research Center of Traditional Chinese Medicine Intelligent Rehabilitation, Ministry of Education, Shanghai, China; ^5^ Department of Rehabilitation Medicine, Tongren Hospital, Shanghai Jiao Tong University School of Medicine, Shanghai, China; ^6^ Yuanshen Rehabilitation Institute, Shanghai Jiao Tong University School of Medicine, Shanghai, China; ^7^ Queensland Brain Institute, the University of Queensland, Brisbane, QLD, Australia

**Keywords:** 40Hz, transcranial alternating current stimulation (tACS), multi-target, stroke, cognitive

## Abstract

40Hz transcranial alternating current stimulation (tACS) has gained attention in cognitive rehabilitation due to its potential to modulate neural oscillations and enhance synaptic plasticity. Most previous studies have focused on single-target stimulation, but post-stroke cognitive impairment (PSCI) involves dysfunction across multiple brain networks. Therefore, multi-target synchronous intervention may offer greater benefits. This case report presents the results of a patient with PSCI who underwent a combined intervention of 40Hz multi-target tACS concurrently with intensive cognitive rehabilitation. The tACS targeted the dorsolateral prefrontal cortex (DLPFC), primary motor cortex (M1), and supplementary motor area (SMA), to address PSCI. Cognitive scales (MOCA; Trail Making Test-A\B (TMT-A\B); Clock Drawing Test; Digit Span Test), sequential reaction time task (SRTT) combined with EEG, transcranial magnetic stimulation (TMS), and magnetic resonance imaging (MRI) were used to evaluate the effects of the intervention. After two weeks of the combined treatment, the patient’s MoCA score improved by 10 points, and the completion time for both TMT-A and B and the reaction time of SRTT was shortened. TMS results indicated reduced resting motor threshold (RMT) and central motor conduction time (CMCT), suggesting increased cortical excitability and enhanced synaptic plasticity. Both EEG and MRI showed increases in activation and functional connectivity in the targeted brain regions, implying improved synchronisation of neural networks. These findings suggest that 40Hz multi-target tACS, when applied as an adjunct to intensive rehabilitation, may be a promising approach for alleviating PSCI.

## 1 Introduction

Post-stroke cognitive impairment (PSCI) affects about 30%-50% of survivors, mainly manifesting by impairments in attention, memory, and information processing speed, which seriously affects the rehabilitation process and quality of life (1). Traditional cognitive rehabilitation training combined with drug therapy has limitations such as large individual differences in efficacy and obvious drug side effects. In recent years, non-invasive brain stimulation techniques (NIBS) based on the principle of neuroplasticity (2), such as transcranial direct current stimulation (tDCS), have shown some potential (3). Still, their limitations in regulating neural network synchronisation have prompted researchers to turn to more frequency-specific transcranial alternating current stimulation (tACS). The 40Hz gamma frequency was selected for its established role in supporting cognitive functions through the modulation of gamma oscillations (4, 5). This rationale is underpinned by evidence demonstrating that 40Hz stimulation can induce synaptic plasticity (6) and enhance neural network connections (7), providing a theoretical basis for clinical translation. as evidenced in neurological entrainment paradigms (8). Consequently, 40Hz tACS was employed to specifically target the patient’s primary deficit in executive function following post-stroke cognitive impairment (PSCI).

Existing studies have mostly used single-target transcranial electrical stimulation (e.g., left dorsolateral prefrontal cortex) (9), ignoring the multi-node damage characteristics of post-stroke cognitive networks. Studies have confirmed that dual-target tDCS can improve cortical excitability and motor learning ability in healthy people more than single-target tDCS (10, 11), and more effectively improve the cognitive function and motor function of Parkinson’s patients (12, 13). Multi-target synchronous stimulation strategies may more effectively reconstruct impaired cross-network functional connections by simultaneously regulating key nodes of the default mode network (DMN) and the central executive network (CEN), such as the prefrontal-parietal cortex. However, the current clinical research on multi-target 40Hz tACS for PSCI is still blank, and its safety, tolerability, and impact on specific cognitive domains need to be explored on a case-by-case basis.

This report records the application process of 40Hz multi-target tACS (dorsolateral prefrontal DLPFC, primary motor cortex M1, and accessory motor area SMA) in patients with PSCI through multi-dimensional cognitive assessment, including MOCA, Trail Making Test-A (TMT-A), Trail Making Test-B (TMT-B), Clock Drawing Test (CDT), and Digit Span Test (DST). Combined with transcranial magnetic stimulation (TMS), electroencephalography (EEG), and magnetic resonance imaging (MRI), the changes in brain activation value and brain network connection value were further analyzed to further analyse the underlying neural mechanism and explore the improvement effect of this intervention on cognitive function, memory function, executive function, and information processing speed.

The results will provide key parameters (such as stimulation duration and target combination scheme) for subsequent randomised controlled trials and provide translational medical evidence for understanding the mechanism of 40Hz nerve oscillations in cognitive rehabilitation, which has important implications for the development of new neuromodulation therapies.

## 2 Case description

A 75-year-old female was admitted with “left limb movement impairment and cognitive dysfunction for 3 months.” On 2025.3.30, she suddenly developed speech arrest, mouth deviation, and left-sided weakness without headache or consciousness disturbance. Initial CT showed right frontal hypodensity. Subsequent MRI (2025.3.31) revealed acute infarcts in left frontoparietal regions and corona radiata. DSA demonstrated right internal carotid artery occlusion distal to ophthalmic artery and M1 segment occlusion with moyamoya collaterals. Despite antithrombotic therapy, infarct expansion was noted on repeat MRI (2025.4.6), prompting conservative management.

Chronic conditions included: Hypertension (30 years, max 190+/90 mmHg, controlled with valsartan/amlodipine); Type 2 diabetes (7 years, HbA1c 6% on empagliflozin/sitagliptin-metformin); Dyslipidemia (on atorvastatin);Previous stroke with residual deficits; Neurological Examination; Intact ocular movements and cranial nerves; Left hemiparesis: Upper limb Medical Research Council (MRC) scale 4-4-3-2 (shoulder/elbow/wrist/hand), lower limb MRC 4-4-4-4; Mild left-sided sensory reduction; Impaired coordination (finger-nose and heel-shin tests); Normal muscle tone (Modified Ashworth Scale 0); Reflex asymmetry: Left biceps/triceps 2+, right plantar equivocal.

Rehabilitation-related assessment content: ① motor function: Brunnstrom Stage: Upper limb IV, hand IV; Fugl-Meyer Assessment: Upper extremity 30, lower extremity 40; Berg Balance Scale: 36; ②Cognitive Function: MoCA: 13; TMT-A: 3’19”39, TMT-B: 3’55”81; CDT: 7/10 (Rouleau’s 10-Point Scale) The CDT was administered and scored according to the widely used and validated 10-point quantitative scoring system developed by Rouleau et al. (14). This standardized method evaluates the integrity of the clock face, the presence and correct placement of all numbers, and the presence and correct placement of the hands, with a total score ranging from 1 to 10 (higher scores indicating better performance). Its reliability and applicability in clinical research have been well-documented (6); DST: Forward 5, Backward 2; SRTT: RT 933.02ms; ③ Neurophysiology: TMS: Resting motor threshold (RMT) 69% MSO, CMCT 15.38ms. Clinical examination and baseline assessment findings are concluded in **Table 1**.

**Table 1:** Clinical examination and baseline assessment findings.

Domain	Assessment tool	Finding/score
Neurological Examination	Cranial Nerves	Intact ocular movements
	Motor Strength (MRC Scale) - Left Side	UL: 4-4-3-2 (shoulder/elbow/wrist/hand); LL: 4-4-4-4 (hip/knee/ankle/toes)
	Sensory Function	Mild left-sided reduction
	Coordination	Impaired (finger-nose, heel-shin tests)
	Muscle Tone (Modified Ashworth Scale)	0 (Normal)
	Reflexes	Left biceps/triceps 2+; Right plantar equivocal
Motor Function	Brunnstrom Stage	Upper Limb: IV; Hand: IV
	Fugl-Meyer Assessment	Upper Extremity: 30; Lower Extremity: 40
	Berg Balance Scale	36
Cognitive Function	Montreal Cognitive Assessment (MoCA)	13/30
	Trail Making Test (TMT)	Part A: 3’19”39; Part B: 3’55”81
	Clock Drawing Test (CDT)	7/10 (Rouleau system)
	Digit Span Test (DST)	Forward: 5; Backward: 2
	Serial Reaction Time Task (SRTT)	Reaction Time: 933.02 ms
Neurophysiology	Transcranial Magnetic Stimulation (TMS)	RMT: 69% MSO; CMCT: 15.38 ms

MRC, Medical Research Council; UL, Upper Limb; LL, Lower Limb; MSO, Maximum Stimulator Output; RMT, Resting Motor Threshold; CMCT, Central Motor Conduction Time.

## 3 Methods

### 3.1 Routine rehabilitation treatment

During the 40Hz multi-target tACS intervention, the patient maintained the original rehabilitation plan in the Rehabilitation Department of Shanghai Seventh People’s Hospital every day, Monday to Friday, 2 times/day, 30 minutes/time, a total of 2 weeks, including: 1) physical therapy (PT): 30 minutes a day, including Brunnstrom stage III. upper limb training (10–15 times/group×3 groups), balance function training (center of gravity shift/incline board training) and gait correction; 2) Occupational therapy (OT): 30 minutes a day, using task-oriented training (e.g., building blocks, simulating the use of tableware) and environmental adaptation training. All daily rehabilitation treatments are carried out by the same rehabilitation team.

### 3.2 40Hz multi-target tACS

**Figure 1:**
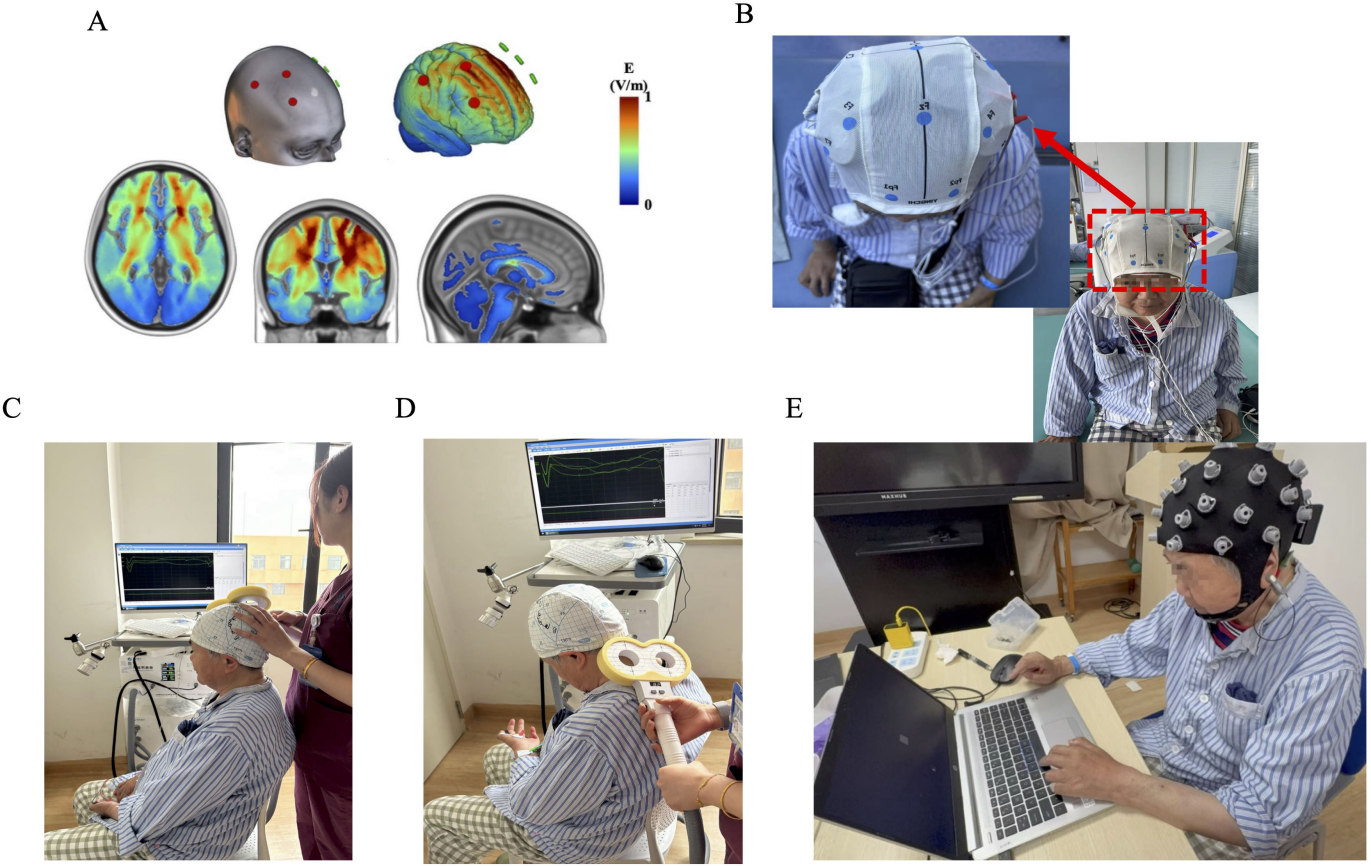
**(A)** The electric field (envelope electric field) intensity in the MNI152 standard head model under multi-target stimulation methods. **(B)** Schematic diagram of wearing multi-target tACS head electrode. **(C)** Schematic diagram of transcranial magnetic stimulation (TMS) evaluation of cerebral cortex motor evoked potential (MEP). **(D)** Schematic diagram of target placement for TMS assessment of central motor conduction time (CMCT). **(E)** Schematic diagram of sequence reaction task time (SRTT) and electroencephalogram (EEG) synchronous acquisition.

By selecting the electrodes required for stimulation and the intensity of the stimulation current in NervioWeb Online Brain Regulation Experimental Platform 1.1.0, the distribution of electric field (envelope electric field) intensity in the MNI152 standard head model under different stimulation methods is simulated and calculated as shown in **Figure 1A**. Using a portable transcranial electrical stimulator (tCE-E2000, Shenzhen Ying Zhi), the tACS session was delivered to the cortex via surface sponge electrodes (diameter of 5cm) soaked in 0.9% NaCl, which were employed and secured in place using a gauze head cover. The anodal electrodes were positioned over the right DLPFC (F4), right primary motor cortex (C4), and the right SMA region (approximately 2 cm anterior to the Cz electrode site along the sagittal midline) according to the international 10–20 EEG system (15). The cathode is placed in the corresponding position of the contralateral brain region. The rationale for simultaneously targeting the right DLPFC, M1, and SMA is based on their synergistic roles within a commonly impaired cognitive-motor network post-stroke. The DLPFC serves as a core hub for executive functions; M1contributes not only to motor execution but also to learning; and the SMA is integral for motor planning and initiation (16). We hypothesize that concurrent 40Hz tACS over this distributed network will enhance gamma-band synchronization and functional connectivity among these nodes, thereby yielding synergistic improvements in post-stroke recovery by modulating the cognitive-motor interface, an effect surpassing that of isolated stimulation. The parameters are set as follows: stimulation frequency 40Hz, sine wave; Peak current 1mA. Schematic diagram of actual tACS operation (**Figure 1B**).

### 3.3 Transcranial magnetic stimulation TMS

Transcranial magnetic stimulation (TMS) figure-eight coil(Xiangyu Medical, China) to assess the resting movement threshold (RMT, **Figure 1C**) (17) and central conduction time (CMCT, **Figure 1D**) (18). Electromyography recorded the evoked potential (MEP) of the right dorsal first dorsal interosseous muscle. The TMS coil is placed tangentially on the scalp area corresponding to the left first-hand motor area, with the coil handle 45° backwards and pointing laterally with the sagittal plane. The recording electrode is attached to the abdomen of the abductor brevis muscle of the right thumb, the reference electrode is placed at the abductor brevis tendon, the grounding electrode is set at the wrist, and the target recording muscle is the abductor brevis muscle of the finger (18). The subject sits comfortably and keeps the head and arms relaxed. An independent evaluator placed the coil in the M1 region of the right brain. The cortical stimulation point was localized as follows: take the intersection of the ear connection and the nasal base-epioccipital trochanter and move 5–7 cm to the left along the ear connection and move forward 1.5 cm (M1 area), the coil and the midbrain line are at a 45° angle, and the handle is facing backward (19). RMT was the lowest stimulation intensity that could induce more than 50 μV MEP waves in 5 out of 10 stimuli. The operator will adjust the intensity of the magnetic stimulation to induce MEPs with an average amplitude of 1mV (peak-to-peak), and perform 5 sequence stimulations at each intensity level, and finally take the average of the 5 MEP waveforms as the data analysis index.

### 3.4 Sequence reaction task combined with EEG

The series reaction time test task (SRTT) is the most used method for assessing motor learning function. Deary-Liewald software was applied in this study (20)To evaluate the changes in motor learning ability before and after tACS intervention. Before starting SRTT, subjects will sit in front of a 15.6-inch computer screen with a long space bar on the keyboard with their left hand on it. A white square will be positioned in the center of the computer screen, contrasting with the blue background. The task is to react to the appearance of diagonal crosses within each square by quickly using the corresponding keyboard keys. Subjects were trained on keystrokes before the official start until the subjects were familiar with the experimental process. During the experiment, the subjects were asked to relax, concentrate, sit upright at 75cm from the monitor, blink less, and keep their heads as still as possible (**Figure 1E**). Participants need to press the keys as soon as possible after each cross appears. The task included 10 practice tests and 80 experimental attempts, and the total experimental time of each subject did not exceed 10 minutes. The stimulus interval (in milliseconds, refers to the duration between a response and the subsequent cross) will be randomly set in the range of 1000 ~ 3000 ms. The computer program records the reaction time and stimulus interval time for each trial. on day 0 (T0); the seventh day of the intervention (T1); Measured on the 14th day of the intervention (T2).

The EEG signal was recorded according to the electrode position of the international 10–20 system (**Figure 1E**). EEG data collection uses the 32-conduct EEG recording and analysis system produced by Zhentai Company, the grounding electrode is AFz, the reference electrode is the left papillae, and the ocular electrode is recorded with Fp1 and Fp2 electrodes. The bandpass filter is 0.01~100 Hz, the sampling rate is 1000 Hz, and the scalp impedance is less than 10 kΩ. In this study, a wireless 32-channel EEG acquisition system (32-channel Zhentai Technology Co., Ltd.) was used to record the EEG activity of the subjects during SRTT (**Figure 1E**). Before starting the experiment, the researcher will provide the following instructions to the participants: (1) ensure that the smallest movements (except for hand button movements), especially the head should not be swayed left or right; (2) Avoid swallowing and other actions when performing button operations; (3) Turn off communication devices, such as mobile phones, or set them to silent or airplane mode to mitigate external interference.

The EEG data were acquired and exported in EDF format, with preprocessing and spectral analyses were performed using the EEGLAB toolbox (https://sccn.ucsd.edu/eeglab/index.php) running in the MATLAB (Version 2016b, The MathWorks, Inc., Natick, MA, United States) environment. The processing flow of EEG power spectral density (PSD) data is as follows: (1) After importing the raw data, filtering (1–30 Hz bandpass filter) is applied, and methods such as ICA decomposition are used to remove artifacts like eye movements and electromyographic (EMG) signals; (2) The cleaned continuous data is then divided into shorter, non-overlapping or overlapping epochs, with window functions (e.g., Hanning window) set for subsequent analysis; (3) The power spectrum is calculated using the spectopo function from EEGLAB or related plugins, performing FFT on each data segment to obtain the PSD estimate for each channel; (4) Finally, absolute or relative power values for standard frequency bands (Gamma-band power 30–50 Hz and 50–80 Hz) are extracted, statistical comparisons are performed, and spectral curves or topographic maps are plotted for visualization.

### 3.5 Resting state functional magnetic resonance fMRI

MRI data were acquired using a 3T Siemens Verio scanner (Siemens, Erlangen, Germany). The scanning sequences were performed as follows: (1) resting functional MRI (rs-fMRI): repetition time (TR) = 2100 ms, echo time (TE) = 30 ms, flip angle = 90°, voxel size = 3.1 × 3.1 × 4.0 mm³, 42 layers of axial section, field of view (FOV) = 200 mm× 200 mm, matrix size = 64 × 64, slice thickness = 4.0 mm, no gap; (2) High-resolution T1-weighted structural image (T1WI): TR = 8.2 ms, TE = 3.2 ms, flip angle = 12°, FOV = 220 mm×220 mm, matrix=256×256, slice thickness=1 mm, yielding a voxel size of approximately 0.9 × 0.9 × 1.0 mm³. The total duration of the scanning session was approximately 25 minutes. The subject was asked to close their eyes but remain awake during the scan, and provided with soundproofing earplugs to reduce noise interference. Before the scan began, the subject was informed to prepare for the scan and instructed to keep their body stable and avoid head movements and limb movements as much as possible.

The spatial preprocessing and analysis procedures performed on imaging data analyses utilized the RESTplus V1.2 (the Resting-State fMRI Data Analysis Toolkit plus V1.2, http://www.rfmri.org) carried out on the MATLAB (Version 2016b, The MathWorks, Inc., Natick, MA, United States) (21). The Data preprocessing consisted of removing the first five time points, slice timing, realignment, reorientation, normalization, smoothing, detrending, nuisance covariates regression, filtering (0.01 Hz-0.08 Hz), and ALFF/Degree Centrality (DC) calculation. Both ALFF and DC values were transformed into Z-scores for group-level analysis. Using MRICron (https://www.nitrc.org/projects/mricron) overlay the pre-processed ALFF and DC maps for each subject. Then, create a composite visualization that highlights brain regions where these values increased post-intervention in red.

## 4 Results

### 4.1 40Hz multi-target tACS improves cognitive function in patients

**Figure 2:**
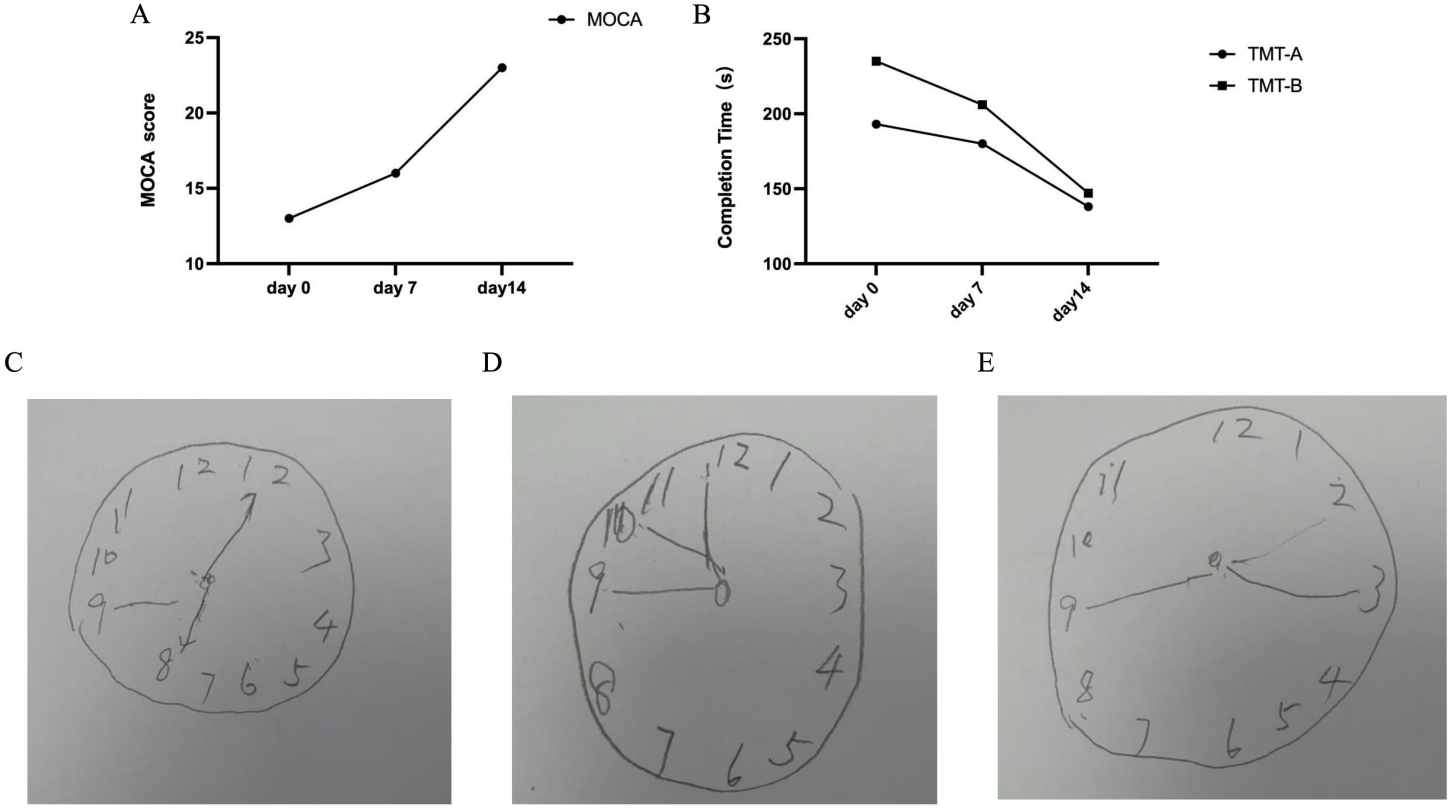
**(A)** The results of Montreal Cognitive Assessment (MOCA, score). **(B)** The results of the Trail Making Test-A/B (TMT-A/B, s). **(C)** The score of the Clock Drawing Test (CDT) at day 0. **(D)** The score of the CDT at day 7. **(E)** The score of CDT at day 14.

The 2-week 40 Hz multi-target tACS increased the score of MoCA in this patient (**Figure 2A**), reduced the completion time of the TMT-A (reduced 28.49%) and TMT-B (reduced 34.77%) as shown in **Figure 2B**, and the results of the Clock Drawing Test (CDT) were shown in **Figures 2C-E** (7-7-8). The improvement effect of the Digit Span Test (DST)results is shown in **Table 2**.

**Table 2:** Test questions for memory breadth.

Test content	Day 0	Day 7	Day 14
First Set Forward Span	5-digit: first attempt wrong, second correct	5-digit: first attempt wrong, second correct	5-digit: first attempt wrong, second correct
Second Set Forward Span	5-digit correct, 6-digit wrong	5-digit correct, 6-digit wrong	5-digit correct, 6-digit wrong
Result	Only passed 5-digit	Only passed 5-digit	Only passed 5-digit
First Set Backward Span	3-digit: both attempts wrong	3-digit: first wrong, second correct	3-digit: first wrong, second correct
Second Set Backward Span	3-digit correct	3-digit correct	3-digit correct
Backward Span Result	Only passed 2-digit	Only passed 3-digit	Only passed 3-digit

### 4.2 40Hz multi-target tACS improves the motor learning ability of patients

**Figure 3:**
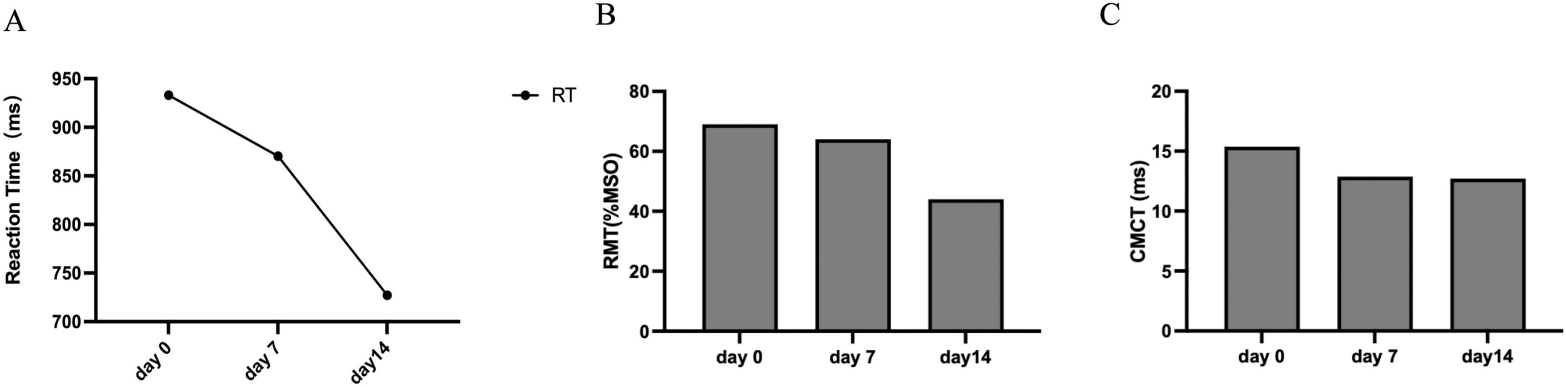
**(A)** The results of sequence reaction task time (SRTT, ms). **(B)** The results of Resting Motor Threshold (RMT, %MSO). **(C)** The results of the Central Motor Conduction Time (CMCT, ms).

The reaction time (RT) in SRTT was shortened by 6.75% and 16.43% on day 7 and day 14, respectively (**Figure 3A**), indicating that the patient’s reaction speed and motor learning ability improved after 14 days of 40Hz multi-target tACS intervention.

### 4.3 40Hz multi-target tACS improves cortical excitability in patients

The TMS results showed that the RMT (**Figure 3B**) and CMCT (**Figure 3C**) of the patient on the first day and day 14 were lower than the baseline, and the decrease was most obvious on the 7th day, indicating that 40Hz multi-target tACS improved the patient’s cortical excitability and nerve conduction efficiency. All the neuropsychological test scores are concluded in **Table 3**.

**Table 3:** A summary table including all neuropsychological test scores.

Neuropsychological test	Day 0	Day 7	Day 14	Notes
MoCA (Total score/30)	13	16	23	Higher scores indicate better function.
TMT-A (second)	193	180	138	Lower times indicate better processing speed.
TMT-B (second)	235	206	147	Lower times indicate better executive function.
CDT (Score/10, Rouleau system)	7	7	8	Higher scores indicate better visuoconstruction and executive function.
DST (Length, Forward Span)	5	5	5	Higher numbers indicate better attention.
DST (Length, Backward Span)	2	3	3	Higher numbers indicate better working memory.
SRTT (Reaction time, milliseconds)	933.025	870.300	727.183	Lower times indicate better procedural learning.
RMT (% MSO)	69	64	44	Lower times indicate higher cortical excitability
CMCT (ms)	15.38	12.87	12.7	Lower times indicate faster central conduction velocity

Data presented as raw scores. MoCA, Montreal Cognitive Assessment; TMT, Trail Making Test; CDT, Clock Drawing Test; DST, Digit Span Test; SRTT, Serial Reaction Time Task; RMT, Resting Motor Threshold; CMCT, Central Motor Conduction Time; ms, milliseconds; % MSO, Percentage of Maximum Stimulator Output.

### 4.4 40Hz multi-target tACS increases the power spectral density of the target brain region

**Figure 4:**
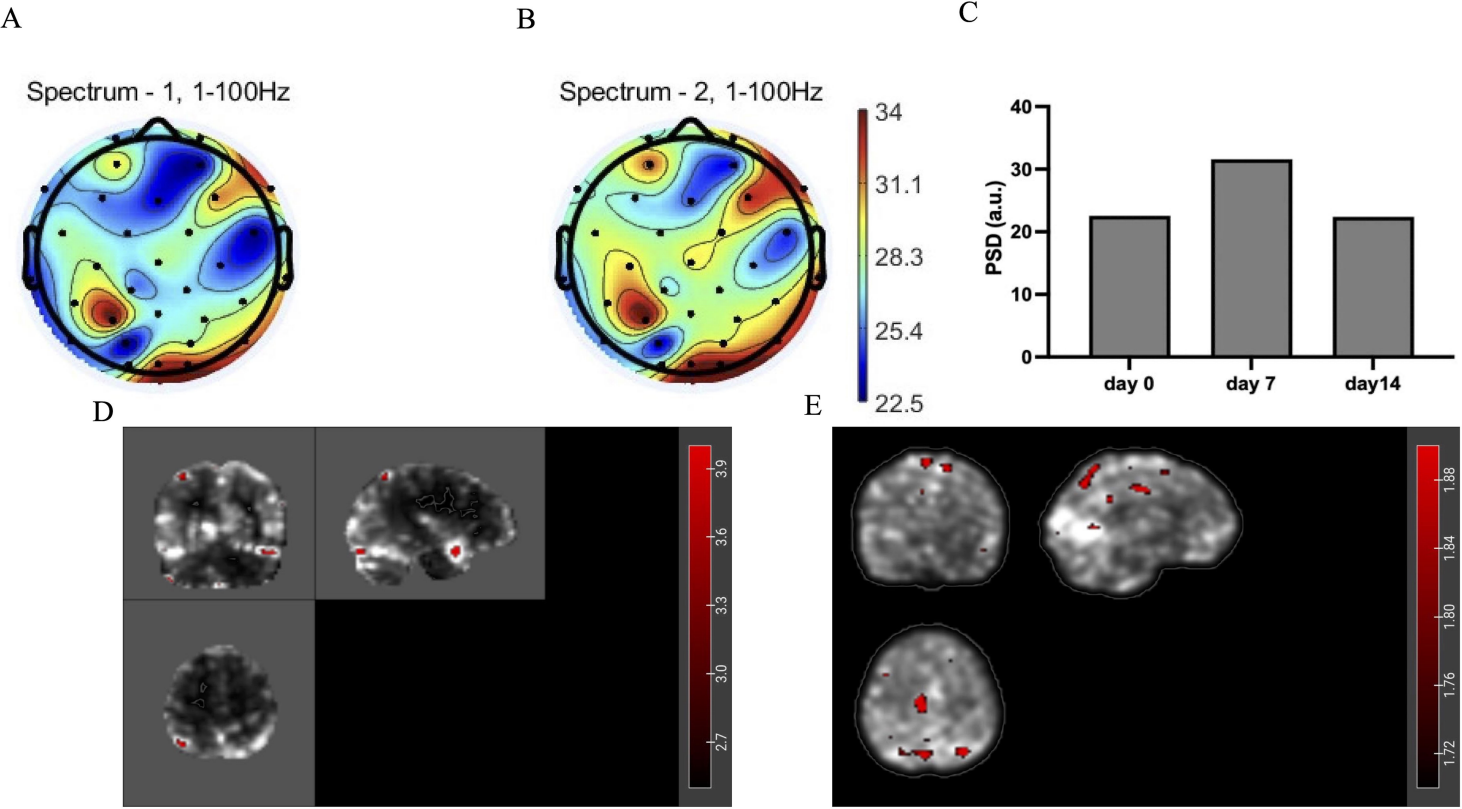
**(A)** Topographic map of gamma-band (1-100Hz) power spectral density (PSD) before intervention (day 0). **(B)** Topographic map of gamma-band (1-100Hz) power spectral density after intervention (day 14). **(C)** The PSD value of slow_gamma (30-55Hz) of brain’s all channels. **(D)** Changes in zALFF values of MRI after 14 days of intervention compared to before (red areas indicate increased values). **(E)** Changes in Degree Centrality (DC) of MRI after 14 days of intervention compared to before (red areas indicate increased values).

EEG results showed that the PSD value of the target brain region (right DLPFC, SMA) increased (**Figures 4A, B**), and the PSD value of slow_gamma (30-55Hz) of all channels increased most after 7 days of intervention (**Figure 4C**) and decreased slightly on day 14. MRI results showed that after 14 days of intervention, the activation value of the prefrontal brain region increased (**Figure 4D**), and the local connection value of the frontoparietal increased (**Figure 4E**). These changes demonstrate modulation of both local activity and functional connectivity patterns following tACS stimulation.

## 5 Discussion

In this study, 40Hz multi-target tACS (DLPFC-M1-SMA combined stimulation) was used for the first time to intervene in patients with post-stroke cognitive dysfunction. The application of 40Hz multi-target tACS was associated with improvements in cognitive scores (the MoCA score increased by 10 points) and executive function (task response speed was accelerated during SRTT), but the improvement of memory was relatively limited, suggesting that tACS may be selective in improving cognitive function. This finding provides a new non-invasive neuromodulation strategy for post-stroke cognitive rehabilitation.

In terms of neural mechanisms, multimodal detection results (TMS/EEG/fMRI) showed that tACS may improve neural network functional connectivity by enhancing gamma band (30–55 Hz) neural oscillations, increasing cortical excitability (manifested by reduced RMT), and promoting prefrontal activation (BOLD signal enhancement). This is consistent with the mechanism by which gamma oscillations promote synaptic plasticity reported in previous studies (22). However, the EEG data at 14 days after the intervention showed a slight decline in efficacy, suggesting that the sustained effects of the current treatment regimen still need to be optimized, stimulation therapy may need to be adjusted, or maintenance intervention strategies may be adopted.

The selection of neuromodulation targets was based on the patient’s specific multi-domain deficits, as identified through our comprehensive diagnostic and assessment workup. First, to ensure the cognitive decline was primarily of vascular origin, other common etiologies, including metabolic disorders, nutritional deficiencies, and major depression, were ruled out via laboratory testing, neuroimaging, and clinical assessment. The rationale for target selection was then directly derived from the patient’s profile: M1 stimulation aimed to address left hemiparesis and poor hand function (evidenced by Brunnstrom and Fugl-Meyer scores); DLPFC stimulation targeted profound executive dysfunction (as indicated by the MoCA, TMT, and DST scores); and SMA modulation was chosen to improve motor planning and cognitive-motor integration, which underpin coordinated movement and daily activities.

Despite this rationale, several limitations must be acknowledged. As a single-case investigation, the findings of this study are inherently limited in their generalizability, and while the observed functional improvements were temporally correlated with the 40Hz tACS intervention, they may have been influenced by concurrent intensive rehabilitation and spontaneous neurological recovery during the subacute phase of stroke—approximately three months post-onset—when the intervention was initiated. The pragmatic study design, which integrated tACS within a conventional rehabilitation program to reflect real-world clinical practice, precludes definitive attribution of the outcomes solely to tACS. Neurophysiological data indicated a transient neuromodulatory effect characterised by a sharp increase in gamma-band PSD that peaked at day 7 but declined to near-baseline levels by day 14, suggesting an acute yet unsustained response that may require ongoing or long-term follow-up in the later stages to verify the effectiveness of stimulation protocols in achieving long-term plasticity.

Despite these limitations, this study provides crucial preliminary evidence supporting the feasibility, safety, and potential benefit of administering 40Hz tACS as a novel adjunct to conventional rehabilitation during a critical recovery window. The promising results underscore the necessity of future sham-controlled trials to conclusively determine the specific additive effect of tACS. Future research can be explored in depth from the following directions: (1) individualized target localization: combined with functional image navigation, to increase precise stimulation of memory-related brain regions (such as the hippocampus); (2) Joint intervention strategy: simultaneous implementation of tACS and computerized cognitive training may produce synergistic effects; (3) Mechanism deepening research: multimodal imaging techniques (such as fNIRS-EEG combination) were used to analyze the dynamic coupling relationship between gamma oscillations and whole-brain functional networks. (4) Long-term efficacy verification: Conduct a multicenter randomized controlled trial (RCT) to evaluate the long-term effects of different treatment regimens on cognitive function.

## 6 Patient perspective

The multi-target tACS intervention was well-tolerated, with only transient scalp tingling reported during sessions. Subjectively, I noticed gradual improvements in attention and short-term memory recall within 2 weeks, particularly during daily activities like medication management. While persistent deficits remained, the treatment provided measurable functional benefits without adverse effects.

## 7 Conclusion

In conclusion, our study provides preliminary evidence that 40Hz multi-target tACS, when combined with intensive rehabilitation, is associated with cognitive improvement in stroke patients, potentially through modulating neurophysiological mechanisms. However, the respective contributions of tACS and conventional therapy cannot be distinguished in this design, necessitating further investigation with sham-controlled protocols.

## Data Availability

The original contributions presented in the study are included in the article/supplementary material, further inquiries can be directed to the corresponding author/s.
